# A Comparative Investigation of Automatic Speech Recognition Platforms for Aphasia Assessment Batteries

**DOI:** 10.3390/s23020857

**Published:** 2023-01-11

**Authors:** Seedahmed S. Mahmoud, Raphael F. Pallaud, Akshay Kumar, Serri Faisal, Yin Wang, Qiang Fang

**Affiliations:** 1 Department of Biomedical Engineering, Shantou University, Shantou 515063, China; 2 Computer and Information Technology Department, IT Institute @ Phoenix College, Phoenix, AZ 85013, USA

**Keywords:** aphasia, deep learning, speech impairment assessment

## Abstract

The rehabilitation of aphasics is fundamentally based on the assessment of speech impairment. Developing methods for assessing speech impairment automatically is important due to the growing number of stroke cases each year. Traditionally, aphasia is assessed manually using one of the well-known assessment batteries, such as the Western Aphasia Battery (WAB), the Chinese Rehabilitation Research Center Aphasia Examination (CRRCAE), and the Boston Diagnostic Aphasia Examination (BDAE). In aphasia testing, a speech-language pathologist (SLP) administers multiple subtests to assess people with aphasia (PWA). The traditional assessment is a resource-intensive process that requires the presence of an SLP. Thus, automating the assessment of aphasia is essential. This paper evaluated and compared custom machine learning (ML) speech recognition algorithms against off-the-shelf platforms using healthy and aphasic speech datasets on the naming and repetition subtests of the aphasia battery. Convolutional neural networks (CNN) and linear discriminant analysis (LDA) are the customized ML algorithms, while Microsoft Azure and Google speech recognition are off-the-shelf platforms. The results of this study demonstrated that CNN-based speech recognition algorithms outperform LDA and off-the-shelf platforms. The ResNet-50 architecture of CNN yielded an accuracy of 99.64 ± 0.26% on the healthy dataset. Even though Microsoft Azure was not trained on the same healthy dataset, it still generated comparable results to the LDA and superior results to Google’s speech recognition platform.

## 1. Introduction

Over the past several decades, strokes have become the leading cause of long-term disability and the second leading cause of death worldwide [1]. There are varieties of functional impairments associated with stroke survivors, such as cognitive, motor, and speech impairments. One recent report stated that over 80 million people suffer strokes worldwide, and one-third of them have aphasia [2].

Patients with aphasia (PWA) are likely to experience impairments in listening, reading, writing, and speaking. In addition, PWAs face communication difficulties, such as difficulty with speech, which may cause experiences of frustration, social isolation, and difficulty performing activities of daily living (ADL). Previous studies have indicated that the early commencement of supervised rehabilitation of PWA leads to a faster recovery [3]. Aphasia is commonly assessed manually with one of the well-known assessment tools, such as the Chinese Rehabilitation Research Center Aphasia Examination (CRRCAE [4]), for Chinese-dialect-speaking patients), the Aachen Aphasia Test (AAT [5]), for German-speaking patients, the Boston Diagnostic Aphasia Examination (BDAE [6]), for English-speaking patients, and the Arabic Diagnostic Aphasia Battery (A-DAB [7]), for Lebanese-Arabic speaking patients. Typically, these tests (referred to as batteries) assess the language function, content, fluency, auditory comprehension, repetition, naming, writing, and calculation. Aphasia rehabilitation depends on assessing people with speech impairments using these subtests, which are used by speech-language pathologists (SLPs). It is, however, labor-intensive to assess aphasic speech manually, which requires the presence of an SLP. Furthermore, SLPs are increasingly having difficulty assessing individual patients with PWA as stroke incidents increase yearly [1]. The development of an automatic method for assessing speech impairments for PWA is, therefore, crucial.

There have been several studies investigating automatic speech assessment for people with aphasia. Using gated recurrent units and CNN algorithms, Qin et al. [8] presented an end-to-end approach to assess Cantonese-speaking PWAs. Using support vector machines (SVMs) and a modified density-based clustering algorithm, in [9], normal from pathological voices were distinguished. The authors in [10] presented a method for detecting aphasia speech using dynamic time-warping algorithms and Mel-frequency cepstral coefficients (MFCCs). CNN and time frequency were used in our previous study [11] to assess impaired speech automatically for Mandarin-speaking aphasic patients. An accurate assessment of the speech severity level was found to correlate with the CNN-based model in twelve aphasic patients [11]. 

However, there is no published research on the automation of aphasia battery subtests using existing, cloud-based, automatic speech recognition platforms, such as Microsoft Azure and Google speech-to-text. These platforms are widely used in daily activities with outstanding performance on healthy speech; therefore, it is worthwhile to investigate their suitability for aphasia assessment. 

In this paper, we compared the performance of the aforementioned off-the-shelf speech recognition platforms to previously investigated custom CNN and linear discriminant analysis (LDA) algorithms within the restricted scope of the aphasia batteries’ naming and repetition subtests involving no spontaneous speech but only isolated, pre-defined words. Both healthy and aphasic speech datasets were used. The comparison is not meant to be absolute in any sense since the custom systems were trained on a small set of isolated words, whereas the off-the-shelf platforms were trained on a very large dataset comprising isolated and continuous speech.

This comparative study will assist the development of aphasia assessment tools as part of aphasia batteries.

## 2. Related Works

Machine learning algorithms are increasingly being used in automatic aphasia assessment. Common tasks include discriminating between normal and aphasic speech, assessing the degree of speech impairment for aphasic patients, and discriminating between various aphasia syndromes. Table 1 summarizes some of these works, listing the type of machine learning used, the main contributions, and performance accuracy when applicable. 

Aphasia batteries such as CRRCAE, Boston Diagnostic Aphasia Examination, and Western Aphasia Battery (WAB) comprise several subtests. Examples of such subtests are spontaneous speech, auditory verbal comprehension, repetition, naming, word-finding, reading, writing, and apraxia. Table 2 shows the key subtests and the potential and possibility of automation for each subtest. 

The naming, repetition, and auditory verbal comprehension subtests can be automated using customized machine learning [8,11,12]. These subtests can be automated using customized speech recognition algorithms thanks to the small dataset size the subtests include. However, subtests such as spontaneous speech require an algorithm to detect words automatically, as well as being trained on a large vocabulary. For example, one of the spontaneous speech questions could be “How are you?”. Patients could respond in many ways, such as “Fine”, “Good”, or “Not bad”. In a sentence completion task, a question such as “Sugar is … ?” could be answered appropriately by “sweet” or “white”. 

**Table 1:** Review of works proposing the use of machine learning for aphasia assessment.

Authors	Type of Machine Learning: Classical Machine Learning (CML), Deep Neural Network (DNN)	Major Contributions, Performance
Järvelin and Juhola, 2011 [13]	CML: k-means, SOM, PNN, k-NN, MLP, Bayes, Disc, Tree	Two aphasia assessment tasks were carried out using speech utterances: (1) to discriminate between healthy and disordered speech and (2) to recognize the patients’ aphasic syndromes. Aphasia datasets from PatLight were used in the first task and naming datasets in the second. In all datasets, none of the ML classifiers appeared to perform exceptionally well. In addition, the selection of a particular classifier should be task dependent.
Kristinsson et al., 2021 [14]	CML: Support Vector Regression (SVR)	The aim was to predict aphasia severity and advise for specific language measures using a multimodal neuroimaging dataset, including task-based functional magnetic resonance imaging (fMRI), diffusion-based fractional anisotropy (FA)-values, cerebral blood flow (CBF), and lesion-load data. According to the authors, different neuroimaging modalities can be integrated to provide a description of how damage to brain tissues and their remaining functionality can affect language function in aphasia.
Qin et al., 2018 [15]	DNN: Time Delay Neural Network Bidirectional Long-Short Term Memory Recurrent Neural Network (TDNN-BLSTM-RNN)	The aim was to predict aphasia severity using speech utterances under the constraint of a lack of training speech data in the intended application domain and the degradation of automatic speech recognition performance for aphasic speech. In our experiment, the predicted severity level and the subjective Aphasia quotient score were highly correlated at 0.842.
Le, 2017 [16]	DNN: i-vectors and multi-task deep Bidirectional Long-Short Term Memory Recurrent Neural Network (BLSTM-RNN)	This Ph.D. dissertation investigated the automatic intelligibility assessment of constrained speech data, specifically the estimation of speech fluidity and prosody. It also investigated aphasic unconstrained speech recognition and then paraphasia detection using BLSTM-RNN. It appeared that there exists a moderate correlation between recognition errors and aphasia severity, which means that automatic speech recognition technology is more suited for non-conversational aphasic speech.
Tsanas et al., 2012 [17]	CML: Support Vector Machines (SVM), random forests	This work investigated how accurately speech signal processing algorithms (dysphonia measures) can predict Parkinson’s disease (PD) symptom severity using speech signals. Experimental results showed that some of the proposed dysphonia measures could complement existing algorithms by maximizing the ability of the classifiers to discriminate healthy controls from PD subjects.
Shahin et al., 2014 [18]	CML: Gaussian Mixture Model-Hidden Markov Model (GMM-HMM) DNN: Deep Neural Network- Hidden Markov Model (DNN-HMM)	The aim was to investigate a pronunciation verification method for use in an automatic assessment therapy tool for child disordered speech. All experiments on normal and disordered speech showed that the hybrid DNN-HMM outperformed the conventional GMM-HMM. A total accuracy rate of 85% was achieved when the system was used with a disordered speech at the phoneme level.
Amami and Smiti, 2017 [9]	CML: Support Vector Machines (SVM) classifier with a Radial Basis Function (RBF) kernel	The aim was to distinguish between normal and pathological voices. The authors used a density-based clustering algorithm named DBSCAN with incremental learning in order to detect noisy samples. They also used MFCC features. The output model was submitted to an SVM classifier to discriminate between normal and pathological voices. Experimental results showed that the method could handle incremental and dynamic voices database, which evolved over time.
Ding et al., 1995 [10]	DNN: Neural Network	The aim was to develop a computer-aided speech therapy system to treat aphasia and articulation disorders in Chinese patients. The authors used MFCCs as features and dynamic time-warping (DTW) algorithms. Real-time speaker-independent non-isolated word recognition was realized successfully. The average recognition rate of 10 numerical numbers was 78%, and that of four words was 77.5%.
Li, 2010 [19]	DNN: Neural Network	The aim was to investigate a computer-assisted speech recognition system for patients with aphasia and dysarthria to help rehabilitate aphasic patients. It used a variety of techniques, including Mel frequency cepstral coefficients (MFCCs) extraction, discrete wavelet transform (DWT), and an artificial neural network. Statistically significant improvements in pronunciation levels were noted after treatment in clinical trials (*p* < 0.025).
Day et al., 2021 [20]	CML: k-means, random forest DNN	This study aimed to investigate how impairment is assessed in aphasic patients and provides clinicians with tools to plan and monitor treatment. The study combined natural language processing (NLP) and regression models to predict severity scores and NLP and classification models to predict severity levels into mild, moderate, severe, and very severe). Their best classification model resulted in an overall accuracy of 73%, with the highest accuracy of 87.5% for mild severity.
Mahmoud et al., 2020 [11]	DNN: Convolutional Neural Network (CNN)	The aim was to assess the severity of impairment in Mandarin-speaking aphasic patients. In their study, the authors found a significant correlation between articulation, fluency, and tone of speech in aphasic patients with different levels of severity. The method used a high-resolution time-frequency distribution (TFD) coupled with a convolutional neural network (CNN). The ML method results and predicted speech impairment levels were found to be significantly correlated in 12 aphasic patients.

**Table 2:** Batteries’ key subtests and suitability of classification algorithms.

Subtest	Task	Description	Classification Models
Spontaneous Speech	Conversational question	Patient verbally responds to personal questions	Off-the-shelf speech recognition platforms (i.e., Microsoft Azure, Google)
	Personal description	Patient describes a picture in the stimulus book	Off-the-shelf speech recognition platforms (i.e., Microsoft Azure, Google)
Auditory Verbal Comprehension	Yes/No questions	Patient must answer personal, environmental, and general questions with a Yes or No. SLP also marks whether the response was verbal, gestural, or through an eye blink	Customized machine learning models (i.e., CNN, LDA)
			Off-the-shelf speech recognition platforms (i.e., Microsoft Azure, Google)
	Auditory word recognition	Patient is shown real objects, as well as cards of pictured objects, forms, letters, numbers, and colors. The patient must point to what the SLP says	Simple computer programming such as multiple-choice selection
	Sequential commands	Patient must execute commands that increase in difficulty and length	Computer vision recognition
Repetition	Words, sentences, and phrases repetition	Patient must repeat words, phrases, and sentences of increasing difficulty	Customized machine learning algorithms require word detection for sentences and phrases
			Off-the-shelf speech recognition platforms (i.e., Microsoft Azure, Google)
Naming and Word Finding	Object naming	Patient must name objects one at a time	Customized machine learning models (i.e., CNN, LDA)
			Off-the-shelf speech recognition platforms (i.e., Microsoft Azure, Google)
	Word fluency	Patient must name as many animals as he/she can in one minute	Off-the-shelf speech recognition platforms (i.e., Microsoft Azure, Google)
	Sentence completion	Patient must complete sentences read to them	Off-the-shelf speech recognition platforms (i.e., Microsoft Azure, Google)
	Responsive speech	Patient must answer sentences read to them	Off-the-shelf speech recognition platforms (i.e., Microsoft Azure, Google)

Off-the-shelf, cloud-based speech recognition platforms are notoriously trained on a large vocabulary and can deal easily with spontaneous speech. It is rational to investigate their suitability for the aphasia assessment task. Since we used Mandarin datasets in this study, we considered two speech recognition platforms under two extreme usage scenarios in China: Microsoft Azure speech-to-text (commonly used) and Google speech-to-text (hardly used). In this paper, we compare the performance of the convolutional neural network (CNN), the linear discriminant analysis (LDA), the Microsoft Azure speech recognition platform, and the Google speech recognition platform over the naming and repetition subtests using healthy and aphasic speech datasets.

## 3. Materials and Methods

### 3.1. Dataset

For this investigation, we used the same dataset as our previous study [11]. However, crucial details about the experiment are mentioned here for coherence. In this study, twelve aphasic patients (including five females) with a mean age of 61.8 ± 14.4 and thirty-four healthy subjects (including 11 females) with a mean age of 21.5 ± 3.1 years participated. The twelve patients were recruited from the Jiaxing Second Hospital in Zhejiang, China, and Shantou University’s First Affiliated Hospital (STU), China. Thirty-four healthy participants were recruited from the STU. A summary of the recruited patients is shown in Table 3. The study was approved by the Ethics Committees of both hospitals. The declaration of Helsinki was followed throughout all experiments. 

A Lenovo B613 recording pen with a sampling rate of 48,000 samples/s, each encoded over 16 bits, was used to capture the speech data of healthy subjects and PWAs volunteers. The speech data were recorded in a stereo mode in the WAV format without any compression and at 1536 kbps. A total of twenty Mandarin words and six Mandarin vowels were uttered by participants in this study. The list of the 20 Mandarin words that related to daily items and activities was taken from the CRRCAE standard [5,11]. Each patient with aphasia repeated vowels and words three times on average, and each healthy participant repeated them five times. A preprocessing step was performed on speech samples to eliminate the silent parts at the beginning and end of each sample. Our analysis also excluded non-quantified samples and samples that were noisy, totaling 4% of the samples. To identify the datasets, separate notations were used; in order to distinguish data pertaining to the vowels and words (26 classes) of healthy participants or aphasic patients, the dataset is designated as ‘vowels + words’ in the following sections. In this paper, we will consider the ‘only words’ dataset, which contains only the speech data (20 classes) of healthy participants or aphasic patients.

### 3.2. Microsoft Azure Speech-to-Text API

Cloud computing is a fairly recent technology that provides access via the internet to a vast array of computing resources, such as storage, database technology, security, virtual machines, analytics, computing, internet-of-things (IoT), and computer vision, among others. They can be grouped into software as a service (SaaS), platform as a service (PaaS), and infrastructure as a service (IaaS). An individual or business only needs a low-specification computer or mobile device to connect to a cloud service and obtain access to these resources via a pay-as-you-go model. While traditionally, a business would be burdened by the purchase or rental of IT infrastructure components (servers, software) and dedicated maintenance staff; cloud computing offsets everything to cloud service providers, who run massive data centers to offer these resources. The pay-as-you-go business model has proven to be well worth the immense investments in cloud infrastructure. The platforms that currently dominate the cloud computing industry are Amazon Web Services (AWS), Microsoft Azure, and Google [21]. 

Microsoft Azure provides a total of more than 200 services, divided into about 20+ categories. One of them is ‘Artificial Intelligence and Machine Learning’, containing both general and specialized machine learning tools [22]. General tools tailored for data scientists make it possible to choose an algorithm and train on very specific data. In contrast, specialized tools gathered in the ‘Cognitive Service’ sub-category are for developers without machine-learning experience, requiring only general knowledge about the data. These services always provide a trained model (using the service’s data, not made available to users) and allow the provision of custom data to refine the training of the model. In some cases, services can be combined to provide a chain solution, such as converting speech to text, translating the text into many languages, and then using those translated languages to obtain answers.

These services provide both (REST) application programming interfaces (APIs) and language-based software development kits (SDKs) requiring knowledge of language programming (such as C#, C++, Java, Python, etc). REST APIs access web services in a simple and flexible way without any processing via HTTP requests (responses come back from the server in the form of a resource which can be anything that is similar to HTML, XML, Image, or JSON) [23]. 

In the speech sub-category, the speech-to-text API is relevant to this work. Additionally, known as speech recognition, it enables the real-time or offline transcription of audio streams into text [24]. It currently supports 139 locales to cater to variations in dialects and phonetics, including fifteen for English, two for Chinese Cantonese, three for Chinese Mandarin, and one for Chinese Wu [25]. In addition to providing feedback on pronunciation accuracy and fluency, it also enables real-time pronunciation assessments [26]. A speech-to-text converter that is ‘out of the box’ in each language uses a universal language model (for this language) as a base model, which is trained on Microsoft-owned data and represents commonly spoken languages. The base model is pre-trained with dialects and phonetics that cover a wide range of domains. By adding additional data to the base model, the user can augment its capability when the audio contains ambient noise or involves a lot of industry and domain-specific jargon. However, this approach has not been followed in this study since the aphasia batteries contain only common words. 

It is worth noting that it is also possible to use the speech service without writing any code, using a real-time speech-to-text tool that is accessible from any browser [27]. 

Since the Microsoft Azure speech-to-text API is proprietary technology, there is no public information available about the type of deep learning that supports it, such as the type of architecture, number of layers, number of neurons, etc. It is very likely that the underlying architecture is constantly evolving as a result of ongoing research efforts carried out by the company. It can also be assumed safely that Microsoft has been able to collect extremely large datasets to train its speech models, including Mandarin since Azure has been in use in China for a long time. 

### 3.3. Google Speech-to-Text API

The Google speech-to-text API currently supports 384 speech models and caters to variations in dialects and phonetics, including forty-eight for English, two for Chinese Cantonese, and four for Chinese Mandarin [28].

A wide variety of use cases can be supported by the API, from dictation to captioning to subtitles and captions. The Google Cloud Console provides developers with complete API functionality, allowing them to perform every API function from within the console, making it easier to integrate the API into their applications. Additionally, this enables developers to customize the speech-to-text model and iterate [29].

There are three main modes for performing speech recognition in the API, namely synchronous (for up to one-minute-long audio data), asynchronous (for up to 8 h long recordings), and stream-based (for real-time recognition tasks such as live audio from a microphone [30]). As an alternative, Google’s speech-to-text service can also be used directly from your browser without having to code [31].

As far as the architecture underpinning the API is concerned, it has tremendously evolved over the last ten years thanks to an intensive research effort. Although the exact details are proprietary technology, Google researchers have regularly reported advances and results in the literature. A fascinating account can be found in [32], showing that, each year, new architectures were developed that further increased quality, from deep neural networks (DNNs) to recurrent neural networks (RNNs), long short-term memory networks (LSTMs), convolutional neural networks (CNNs), recurrent neural networks-transducers (RNN-T), and more. The latest research is advanced enough to have produced a prototype (soon to be deployed commercially) of a neural model that is compact enough to fit on a single smartphone and is able to carry out speech-to-text independently from the cloud [32]. 

In terms of datasets, Google products have not been in much use in China. Therefore, it is expected that Google has not been able to collect Mandarin speech datasets as large as its rivals. 

### 3.4. Deep Neural Network Framework

Convolutional neural networks (CNN) with high-resolution TF image inputs were compared to the Microsoft Azure and Google speech recognition platforms. The CNN models are widely used across various applications and domains, including aphasia assessment tasks [9]. In this study, the hyperbolic T-distribution (HTD) [33,34,35] was used as a TF-based image input to the CNN model within each model. The HTD has been found to produce a high-resolution TF image of Mandarin speech signals; hence, it can improve speech signal classification when used with the CNN model [11,36,37]. 

In HTD, the continuous TFD of the analytic signal z(t) associated with the original real signal s(t) can be given as follows [23,33]:

(1) $$\rho \left(t,\;f\right)=F_{\tau \to f}\left[G\left(t,\;\tau \right){*}_{\left(t\right)}K_{z}\left(t,\;\tau \right)\right]$$

where $K_{z}\left(t,\tau \right)=z\left(t+\tau /2\right)z^{*}\left(t-\tau /2\right)\;$is the instantaneous autocorrelation product, F is the Fourier transform, G(t, τ) is the time-lag kernel, and ${*}_{\left(t\right)}$ denotes time convolution. The kernel for the HTD is given by [21,35]

(2) $$G\left(t,\;\tau \right)=R_{\sigma }\left(t\right)=\frac{k_{\sigma }}{{\cos h}^{2\sigma }\left(t\right)}$$

where σ is a real positive number and $k_{\sigma }$ is a normalization factor given by:

(3) $$k_{\sigma }=\int_{-\infty }^{\infty }\frac{1}{{\cos h}^{2\sigma }\left(t\right)}dt=\frac{\Gamma \left(2\sigma \right)}{2^{2\sigma -1}\Gamma^{2}\left(\sigma \right)}$$

in which Γ represents the gamma function. 

The performance of a speech recognition algorithm relies on the accuracy of the chosen machine learning method. Therefore, in this section, a comparison between six state-of-the-art CNN architectures over healthy vowels + words and the healthy-only words datasets was introduced to assist in the selection of a suitable CNN architecture. The pre-trained CNN architectures are AlexNet, ResNet-18, ResNet-34, ResNet-50, VGG16, and VGG19 CNN. 

**Figure 1:**
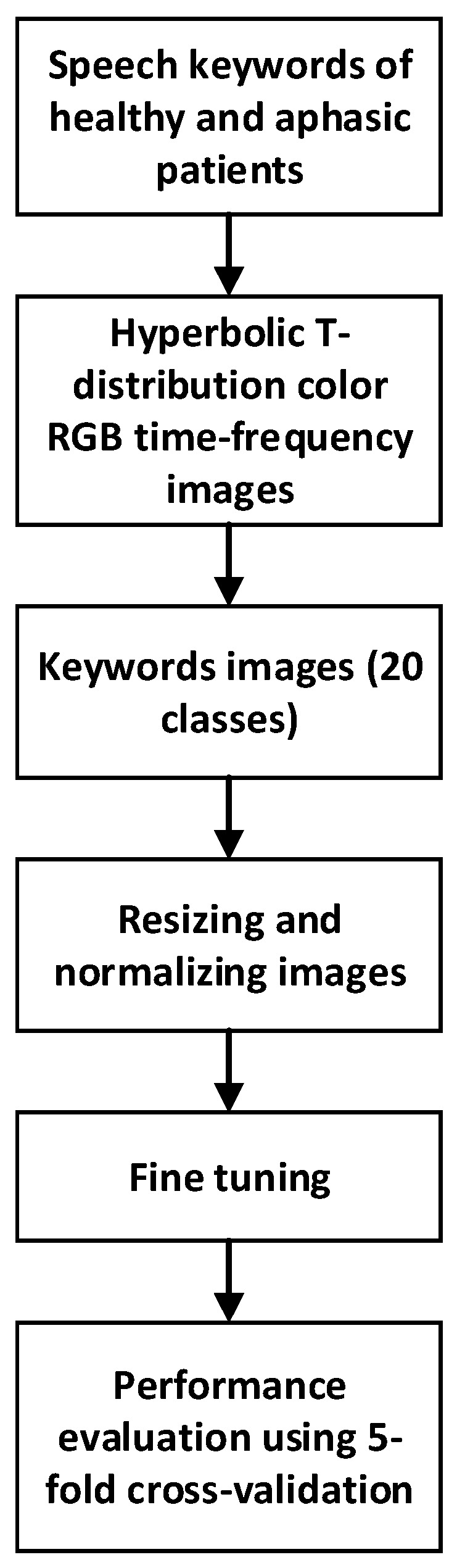
Performance evaluation of the CNN model which utilizes hyperbolic T-distribution RGB color time-frequency images.

Because of the lack of large speech datasets, transfer learning (TL) was utilized in this study to train the CNN models. Considering the characteristics of the ImageNet dataset [38], all TFD RGB color images were resized to 224 × 224 × 3 pixels before feeding them to the pre-trained CNN models. A cyclical learning rate of 0.003 was used for fine-tuning the pre-trained models using the TFD image datasets [39]. For training, a cross-entropy loss function was used along with the ADAM optimizer with default parameters β1=0.9 and β2=0.999 [40]. The weight decay was incorporated with a multiplying factor of 0.01, which was empirically chosen to prevent overfitting [41]. The models were trained using a batch size of 128, with a total of 15 epochs for each model. Using five-fold cross-validation, the classifiers were evaluated for their ability to classify speech data (see Figure 1). The performance metrics used were accuracy and F1-score. Model training was performed using an NVIDIA Tesla P40 GPU, and development was performed using Fastai: a PyTorch-based deep neural network library [42]. For statistical significance evaluations, the Wilcoxon signed-rank test (Exact method) was used. IBM SPSS Statistics 26 was used for all statistical analyses.

Table 4 shows the healthy participants’ performance comparison results for the six CNN architectures. All model results shown in Table 4 are averages of the five-fold cross-validation. The VGG16 model showed the highest accuracy and F1-score, i.e., 99.75 ± 0.1%, for the healthy participants’ only words dataset. For the healthy participants’ vowels + words, ResNet-50 showed the highest accuracy and F1-score, i.e., 98.18 ± 0.57%. For the vowels + words healthy participants’ dataset, ResNet-50 was observed to have a statistically significantly (*p* < 0.05) higher accuracy and F1-score than that of VGG16. Hence, the CNN ResNet-50 architecture was used to compare against Microsoft Azure and Google speech recognition platforms.

## 4. Results

In this section, an evaluation of two customized machine learning algorithms (CNN and LDA) and two off-the-shelf speech recognition platforms (Microsoft Azure and Google) was provided for three scenarios.

For the first scenario, the four speech recognizers were trained and tested on a healthy dataset. For the second scenario, they were trained on healthy data but tested on aphasic data. For the last scenario, an aphasic speech dataset was used both for training and testing. This scenario did not apply to Microsoft Azure and Google speech-to-text platforms since they were pre-trained using healthy speech data.

Standard and well-known performance evaluation metrics were used, namely, accuracy, precision, recall, and F1-score [11].

### 4.1. Machine Learning Algorithms Performance on Healthy Dataset

This section presents the comparative performance of the four speech recognizers using the healthy subjects’ dataset. A total of 20 Mandarin words (yielding 20 classes) embodying everyday objects and activities were used in the training and testing of these algorithms. The words were taken from the CRRCAE battery and belonged to the naming and repetition subtests. 

**Figure 2:**
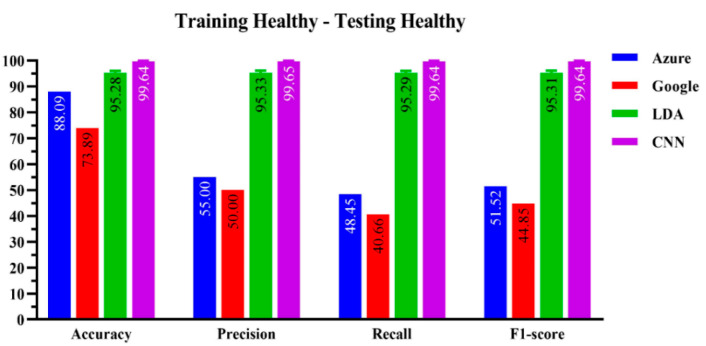
Performance evaluation of the four-machine learning-based algorithms on the healthy subjects’ dataset.

Figure 2 compares the performance of the four speech recognizers. Whatever the performance indicator (PI), the ResNet-50 CNN algorithm using HTD time-frequency (TF) images as the input scored higher than the three other algorithms. In terms of accuracy, the CNN algorithm hit 99.64 ± 0.26%, whereas the LDA scored a lower 95.28 ± 0.79%. The Microsoft Azure speech recognition platform performed quite well with an accuracy of slightly over 88%, far above its Google rival, scoring disappointingly below 75%.

### 4.2. Machine Learning Algorithms Performance on the Joint Healthy-Aphasic Dataset

The comparative performance of the four speech recognizers is presented in this section based on the joint healthy-aphasic dataset. A set of 20 common Mandarin words were used to train these algorithms on healthy speech and test them on aphasic speech.

**Figure 3:**
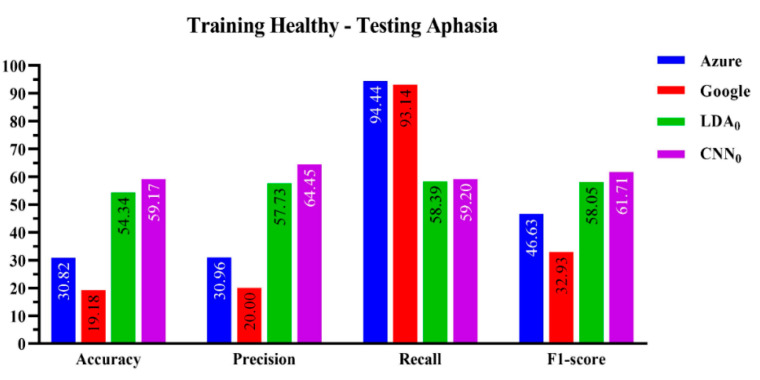
Performance evaluation of the four-machine learning-based algorithms on the joint healthy-aphasic dataset.

Figure 3 compares the performance of the four speech recognizers. The ResNet-50 CNN speech recognition algorithm achieved the highest accuracy, precision, and F1 score.

The accuracy performance was 59.17 ± 0.003% for CNN versus 54.34 ± 0.79% for the LDA. Both off-the-shelf speech recognition platforms performed poorly, scoring below 31%.

### 4.3. Machine Learning Algorithms Performance on Aphasic Dataset

The performance of the CNN and LDA algorithms on the aphasic dataset is compared in this section. A set of 20 common Mandarin words were used both to train and test these algorithms. The Microsoft Azure and Google speech-to-text platforms had been re-trained using healthy speech data and, as such, were not included in this scenario.

**Figure 4:**
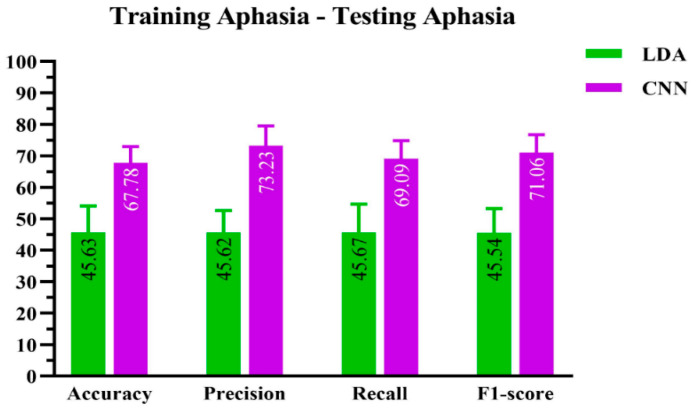
Performance evaluation of CNN and LDA machine learning-based algorithms on the aphasic dataset.

The performance results for CNN and LDA are shown in Figure 4. 

Whatever the performance indicator (PI), the ResNet-50 CNN algorithm scored higher than the LDA algorithm.

As far as accuracy was concerned, the CNN algorithm achieved 67.78 ± 0.003%, whereas the LDA algorithm obtained 45.63 ± 0.79%.

## 5. Discussion

The use of speech recognition platforms, such as Microsoft Azure and Google, has become increasingly common in language learning and speech-to-text dictation. Although several studies have explored the automatic speech impairment assessment of patients with aphasia (PWA) [8,11,12,43], there have been limited applications of off-the-shelf speech recognition platforms for aphasia. The paper has examined the potential of these platforms for the assessment of PWAs in comparison to deep learning-based speech recognition algorithms. 

The results showed that the training and testing datasets have a substantial impact on the performance of machine-learning-based speech recognition algorithms, regardless of whether they are off-the-shelf platforms or customized algorithms such as CNN and LDA. Furthermore, over the three scenarios of speech recognition, the CNN-based algorithm outperformed the other three machine learning algorithms. In the following subsections, we discuss the findings for each scenario. 

### 5.1. The Healthy Dataset Scenario

In this scenario, the healthy subjects’ dataset consisting of 20 isolated words was used to train and test the four machine learning algorithms. The off-the-shelf speech recognizers were pre-trained by Microsoft and Google using healthy speech data, different from the healthy dataset used in this study, to train the customized algorithms. As per the automatic speech impairment assessment (ASIA) procedure described in [11], healthy speech is considered the ideal/standard speech, and aphasic speech was compared against healthy speech for ASIA. Thus, classifying healthy speech with maximum accuracy carries the utmost importance for ASIA. 

The ResNet-50 CNN model with the HTD TF images as input outperformed not only the LDA but also the other two speech recognition platforms. It was expected that it would outperform LDA since the CNN-based classifier has access to unique features from high-resolution images. As for the outperformance of off-the-shelf speech recognizers, it should be borne in mind that those dealing with a very large number of classes (much more than the 20 classes the CNN classifier dealt with). 

A comparison of Microsoft Azure and the Google speech recognition platform shows that they did not fare equally. Clearly, Microsoft Azure displayed a superior performance. This is most likely due to the fact that Microsoft Azure is commonly used in China. 

The advantage of the off-the-shelf speech recognition platforms over the customized ones is that the off-the-shelf platforms were trained on very large language vocabulary/classes. As a result, unlike the CNN-based classifier, they can detect and transcript real-time spontaneous speech consisting of complex sentences. This feature is essential to automating spontaneous speech and word fluency subtests in aphasia assessments, as per Table 2. 

To summarize, in this scenario, the CNN-based classifier would be best for recognizing isolated words (as per some of the aphasia battery subtests in Table 2). However, for spontaneous speech, Microsoft Azure would be the preferred choice.

### 5.2. The Joint Healthy-Aphasic Dataset Scenario

In this scenario, the healthy subjects’ dataset was used to train the classification algorithms, while they were tested using the aphasic speech dataset. Note that the off-the-shelf speech recognition platforms were pre-trained with healthy speech data from a different source.

All algorithms exhibited degraded performance in the form of low accuracy, precision, recall, and F1-score: much lower than in the first scenario. This is something positive, as this will form the basis for discriminating between healthy and aphasic speech and possibly assess impairment severity levels.

It can be observed that the CNN-based classifier consistently outperformed the LDA algorithm and the two off-the-shelf speech recognition platforms.

Machine learning algorithms can be trained using healthy datasets to assess an aphasic patient’s degree of severity in terms of impairment. In this scenario, the classification problem can be transformed into a regression problem by mapping the classifiers or platform outputs to the severity levels’ ground truth [11]. As a result, speech samples from healthy subjects were effectively classified by a model with its highest accuracy of 99.64 ± 0.26. On the other hand, if aphasic speech samples are fed to a similar model, it classifies them as low scoring based on the level of severity. There is a strong correlation between the patients’ severity levels of speech impairment and the CNN model’s final node activations, according to [11]. Two of the recruited patients [11] with different impairment severity levels were able to speak the Mandarin verb chuan1 yi1. When the CNN was activated at the true class node (called ‘normalized true-class output activation (TCOA)’ in [11]), the output activation was 0.35 for the patients with high severity levels and 0.73 for patients with low severity levels. This CNN-based model is appropriate for discriminating between normal and aphasic speech due to the wide range of severity levels among patients.

The speech-to-text API platforms from Microsoft Azure and Google both showed similar behaviors for these two recruited patients [11]. Specifically, the patient with the low severity level had a speech recognition rate of 53.33% and 21.67%, using Microsoft Azure and Google platforms, respectively, whereas the patient with the high severity level had a speech recognition rate of merely 1.33% and 0% using the two platforms, respectively. It is worth noting that it is possible to configure both Microsoft Azure and Google speech-to-text APIs to obtain a value of accuracy or confidence level for individual words in a transcription [44,45], although this has not been conducted in the present study. It is expected that lower confidence levels (yielded for degraded speech) would correlate highly with the patients’ impairment severity levels. This opens the possibility of mapping the confidence level produced by Microsoft Azure and Google speech-to-text APIs to the patients’ impairment severity level, even for the spontaneous speech subtest of the aphasia battery (Table 2). 

It is also possible to discriminate between healthy and aphasic speech (binary classification problem) by adding a decision logic associated with a cut-off threshold at the output of the classifiers [9,46]. The lowest classification cut-off threshold for the two customized classification models to discriminate between healthy and aphasic speech is 0.7. With this classification threshold, the two customized models can achieve an accuracy of 100% to discriminate between healthy and aphasic speech. 

As for the two off-the-shelf speech recognition platforms, if they are configured so that confidence levels are produced for each word of the transcription, it would also be possible to set a cut-off threshold to discriminate between healthy and aphasic speech, both for the spontaneous speech and word fluency subtests of the aphasia battery.

### 5.3. The Aphasic Dataset Scenario

The aphasic speech dataset was used to train and test both CNN and LDA machine learning algorithms. The two off-the-shelf speech recognition platforms were excluded from this scenario since they had been pre-trained with healthy speech data. Similarly to the previous scenarios, the CNN-based algorithm with the HTD TF images as input outperformed the LDA algorithm. Both CNN and LDA exhibited significantly poor performance on the aphasic patients’ dataset. The degradation in performance was due to the diversity of the aphasic dataset [11]. There were differences in the severity levels of speech impairment among the recruited patients, as reported in [10], leading to complex and unresolvable common features. In addition, the datasets of aphasic patients are scarce and often small [11] since there are multiple aphasia types and multiple severity levels. This finding agrees with what is reported in the literature, where data scarcity [47], abnormal speech patterns [48], and speaker variability [49] are challenges to any classification problem. 

## 6. Conclusions and Future Work

In this paper, the performance of the convolutional neural network (CNN), the linear discriminant analysis (LDA), and off-the-shelf speech recognition platforms over the naming and repetition aphasia’s subtest using healthy and aphasic speech datasets have been investigated. The off-the-shelf speech recognition platforms were Microsoft Azure and Google. Microsoft Azure speech-to-text is commonly used in China, in sharp contrast to Google. 

Speech data recorded from twelve aphasic patients and thirty-four healthy subjects, consisting of twenty different Mandarin words, formed the datasets for three scenarios: training and testing on healthy speech; training on healthy speech and testing on aphasic speech; and training and testing on aphasic speech.

The results showed that the CNN-based speech recognition algorithm outperformed the LDA, Microsoft Azure, and Google speech recognition platforms over the three modeling scenarios, that is to say, even when the testing data differed from the training data. Turning the automatic speech recognition problem into an image classification problem via the use of a CNN operating on high-resolution time-frequency images permitting the automatic detection of important speech features led to better classification results than the conventional LDA. This should not hide the fact that, from the point of view of developing automatic methods for assessing speech impairments for PWA, CNNs have the drawback of involving a significantly higher programming effort. 

Another result is that Microsoft Azure outshone Google among the off-the-shelf platforms. This was expected but also calls for the assessment of other off-the-shelf automatic speech recognition platforms.

In the future, the following three directions of development will be pursued.

First, further aphasic speech data collection will be required to cater to the current scarcity of data across various aphasia types (such as Global aphasia, Broca’s aphasia, Wernicke’s aphasia, and amnesic aphasia). This will help improve the accuracy of the CNN-based aphasia detection and discrimination of aphasia syndromes.

Secondly, tools for subtests involving spontaneous speech will be designed. Off-the-shelf speech recognition platforms can be used to automate multiple aphasia subtests due to their ability to detect words within complex sentences. Moreover, these platforms are trained on very large vocabulary datasets, which enable them to transcript spontaneous speech in real-time. Additional performance enhancements should be achieved by retraining them over aphasia datasets. Likewise, our CNN model will be retrained on a much larger vocabulary dataset.

Thirdly, the design of an ensemble classifier will be carried out, which is an augmented classification framework that has the potential to harness the benefits of multiple classifiers. The CNN-based speech recognition algorithm and the Microsoft Azure speech-to-text platform will be key parts of this hybrid system. Ensemble learning methods, including bagging, stacking, and boosting, will be investigated.

## Figures and Tables

**Table 3:** Aphasic patients’ details.

Number of Patients	Gender Male/Female	Age, Yrs. (Mean ± SD)	Cardinal Symptom (#)	Native Dialect (#)
12	7/5	61.8 ± 14.4	Broca (6) Dysarthria (3) Anomic (1) Combined (1) Transcortical motor (1)	Mandarin (6) Teochew (2) Jiaxing (4)

**Table 4:** Performance evaluation of six state-of-the-art CNN architectures for the classification of only words (20 classes) and vowels + words (26 classes) healthy datasets in terms of accuracy and F1-score using five-fold cross-validation.

Model	HTD (Only Words)		HTD (Vowels + Words)	
	Accuracy	F1-Score	Accuracy	F1-Score
AlexNet	98.76 ± 0.29	98.76 ± 0.29	95.33 ± 0.47	95.35 ± 0.46
ResNet-18	99.59 ± 0.19	99.59 ± 0.19	97.55 ± 0.31	97.53 ± 0.32
ResNet-34	99.70 ± 0.20	99.70 ± 0.20	97.97 ± 0.27	97.97 ± 0.26
ResNet-50	99.64 ± 0.26	99.64 ± 0.26	98.18 ± 0.57	98.19 ± 0.57
VGG16	99.75 ± 0.10	99.75 ± 0.10	97.70 ± 0.23	97.70 ± 0.23
VGG19	99.70 ± 0.28	99.70 ± 0.28	97.91 ± 0.15	97.91 ± 0.15
Average	99.52 ± 0.41	99.52 ± 0.41	97.44 ± 1.03	97.44 ± 1.02

## Data Availability

The data presented in this study and the implementation source code are available on request from the corresponding authors.
